# Thyroid hormone use in clinical practice by Israeli endocrinologists: a THESIS* questionnaire survey: *Treatment of hypothyroidism in Europe by specialists: an international survey

**DOI:** 10.1186/s13044-024-00219-2

**Published:** 2025-03-25

**Authors:** Liat Sasson, Keren Kaminer, Chagit Adler Cohen, Laszlo Hegedüs, Roberto Negro, Endre V. Nagy, Enrico Papini, Petros Perros, Roberto Attanasio, Eyal Robenshtok

**Affiliations:** 1 Endocrinology and Metabolism Institute, Rabin Medical Center, Petah-Tikva, 49100 Israel; 2 Sackler Faculty of Medicine, Tel-Aviv University, Tel Aviv, Israel; 3 Department of Endocrinology, Odense University Hospital, Odense, Denmark; 4 Division of Endocrinology, V. Fazzi Hospital, Lecce, Italy; 5 Division of Endocrinology, Department of Medicine, Faculty of Medicine, University of Debrecen, Debrecen, Hungary; 6 Department of Endocrinology and Metabolism, Ospedale Regina Apostolorum - Lifenet Group, Rome, Italy; 7 Institute of Translational and Clinical Research, Newcastle University, Newcastle upon Tyne, UK; 8 Associazione Medici Endocrinologi Scientific Committee, Milan, Italy

**Keywords:** Hypothyroidism, Levothyroxine, Liothyronine, Questionnaire

## Abstract

**Objective:**

Several thyroid hormone formulations are available for treatment of hypothyroidism. This study aimed at evaluating the use of these treatment options by Israeli endocrinologists in various clinical scenarios.

**Methods:**

Israeli Endocrine Society members were invited to participate in a web-based questionnaire, Treatment of Hypothyroidism in Europe by Specialists: An International Survey.

**Results:**

99.2% of respondents used LT4 tablets as first line therapy for hypothyroidism. Thyroid hormone replacement options considered by respondents included LT4 tablets (100%), soft-gel capsules (4.0%), liquid solution (15.4%), combined LT4 + LT3 (2.4%) and LT3 tablets (17.8%). In cases of impaired absorption or persistent symptoms, most would continue LT4 tablets (86.1% and 95.1%, respectively), of whom 39.0% noted that only tablets are available in Israel. In patients with normal serum TSH and persistent symptoms, 95.1% would continue LT4 tablets, 57.5% would consider the addition of LT3 whereas 24.4% stated that LT4/LT3 combination should never be used. In euthyroid patients, LT4 therapy was considered in infertile women with high levels of thyroid antibodies (33.6%) and for simple goiter growing over time (11.4%).

**Conclusions:**

In Israel, LT4 tablets are the treatment of choice for hypothyroidism in most clinical scenarios, including in patients with impaired absorption or with persistent symptoms, for whom a combination therapy with LT4 + LT3 is considered by half of respondents. Other LT4 formulations are not widely available in Israel, thus are infrequently considered compared to other European countries. These data suggest that international guidelines regarding the use of various thyroid hormone formulations in specific clinical scenarios are warranted.

## Introduction

Hypothyroidism is one of the most common endocrine disorders, affecting approximately 10% of the global population, with an estimated prevalence of 3% in Europe [1]. Levothyroxine (LT4) formulations for the treatment of hypothyroidism have evolved over the last decades. While tablets have been the standard, and often the only treatment option for many years, novel formulations, including soft-gel capsules and liquid solutions, were developed to overcome some of the bioavailability issues in tablets [2]. These formulations may have a potential clinical benefit, since the bioavailability of tablets may be impaired when administered with food, medication (e.g., proton-pump inhibitors, calcium and iron supplements), or some gastrointestinal conditions (e.g., celiac disease, bariatric surgery, or atrophic gastritis) [3].

Other preparations for the treatment of hypothyroidism that are used sporadically include desiccated thyroid extract (DTE), which is derived from the thyroid glands of animals and contains both T4 and T3, combined L4 + LT3 preparations, and LT3 monotherapy. While LT4 is the accepted standard therapy in hypothyroid patients according to current guidelines, recent publications point to increasing use of combination therapy with LT4 + LT3 or DTE, driven mostly by anecdotal evidence and increasing demand by patients [4]. According to the European Thyroid Association (ETA) guidelines and other publications, a trial of LT4 + LT3 combination therapy may be considered in hypothyroid patients who have persistent symptoms despite normal serum TSH with LT4 therapy [5, 6]. In the meantime, ample evidence has accumulated showing that LT4 + LT3 combination therapy is not superior to LT4 in terms of quality of life and other patient outcomes [4].

In Israel, LT4 tablets are among the most prescribed medications, as they are the only LT4 preparation readily available for the treatment of hypothyroidism, in comparison to other European countries where other formulations of LT4 and LT3 are available.

The survey was part of an international project referred to as THESIS (Treatment of Hypothyroidism in Europe by Specialists: An International Survey). In light of the limited treatment options in Israel, we aimed to investigate the use of thyroid hormones for hypothyroid and euthyroid patients by Israeli endocrinologists.

## Methods

The questionnaire was developed to evaluate attitudes of European thyroid experts regarding the treatment of hypothyroidism and use of thyroid hormones in euthyroid individuals. The survey was developed in English to ensure comparability between the participating European countries. Survey completion took 5–10 min. The open-access survey platform Google Forms was used to build and distribute the questionnaire, and a written introduction preceded the survey. Nine questions about demographic data (section A) were followed by sixteen questions about treating hypothyroid and euthyroid patients (section B) [7].

An e-mail and a WhatsApp message with an electronic link leading to the voluntary and anonymized questionnaire was sent to all members of the Israeli Endocrine Society (IES) on 27th February 2020, followed by three reminders as well as personal communication between February and May 2020. Thereafter the survey was closed. Survey responses were collected and stored electronically by Google Forms, where the data were accessible by password. Data from the Israeli survey were compared to other countries within the THESIS framework.

### Statistical analysis

Descriptive statistics were used for responses to all questions. Only respondents who had completed all questions about demographic data were considered valid for statistical analysis. In all analyses, respondents stating that they did not know the answer to a given question were pooled in the category with respondents who did not provide an answer. The goodness of fit χ^2^-test was used to compare frequencies between the categorical variables. Pearson’s χ^2^-test was used to test if variables in the demographic data (section A) were independent of the outcome in questions in section B. If any variable was not independent of the outcome in any question in section B, a logistic regression analysis was performed. A two-sided p value of < 0.05 was considered statistically significant. All analyses were conducted using IBM SPSS statistics software version 25 (SPSS Inc., Chicago, IL, USA).

## Results

### Sample characteristics

A total of 123 physicians out of 197 invited from the Israeli Endocrine Society (62.0%) participated in the survey, of whom 116 responded to all questions. Most respondents were women (57.7%), 65.1% were between the age of 41 to 60 years, and 92.7% had more than 10 years in medical practice (Table 1). Endocrinologists and pediatric endocrinologists comprised 85.4% and 11.4% of respondents, respectively, while two internal medicine physicians and two surgeons participated too (1.6% each).

76% of respondents stated that they treated hypothyroid patients on a daily basis, 22.0% on a weekly basis, and 1.0% rarely. Almost all respondents treated more than 50 hypothyroid patients per year (94.3%), and 68.3% treated over 100 patients per year.

**Table 1:** Characteristics of the 123 Israeli respondents

	*n* (%)
Gender	
Female	71 (57.7%)
Male	52 (42.3%)
**Age (years)**	
31–40	14 (11.4%)
41–50	52 (42.3%)
51–60	28 (22.8%)
61–70	18 (14.6%)
70+	10 (8.1%)
Missing data	1
**Years in medical practice**	
0–10	9 (7.3%)
11–20	53 (43.1%)
2 1–30	31 (25.2%)
31–40	20 (16.3%)
40+	10 (8.1%)
**Specialty**	
Endocrinology	105 (85.4%)
Pediatric endocrinology	14 (11.4%)
Internal medicine	2 (1.6%)
Surgery	2 (1.6%)
**Place of employment**	
University affiliated hospital/clinics	77 (62.6%)
Health Maintenance Organization / private clinics	46 (37.4%)
**Number of hypothyroid patients treated per year**	
10 to 50	5 (4.1%)
51–100	32 (26%)
> 100	84 (68.3%)
Rarely	2 (1.6%)

### LT4 formulation preferences

95% of respondents claimed to have control over the LT4 formulation dispensed for their patients, and 99.2% chose LT4 as the first line of therapy. Regarding the type of LT4 formulation prescribed by the respondents, they were allowed to choose more than one option: 100% of them chose LT4 tablets, 4.0% soft-gel capsules, 15.4% liquid solution. Notably, only 2.4% of respondents considered the use of combined LT4 + LT3 as first line of treatment for hypothyroid patients, while 17.8% would only use LT4 + LT3 in specific clinical scenarios. Comparing respondents who used LT4 tablets only with those who used at least one other formulation, no differences were found in years in practice (*p* = 0.2), age (*p* = 0.17), or gender (*p* = 0.6). After initiation of LT4 replacement therapy, most respondents stated that they would re-check serum TSH after 4–6 weeks (70.7%), or 8.

weeks (24.4%). Following a switch of formulation or dose, most respondents would wait 4–6 weeks (77.2%) or 8 weeks (22.8%) before re-checking serum TSH.

**Table 2:** Use of LT4 formulations

	Tablets	Soft-gel capsules	Liquid solution	No change with formulations	Only tablets available in Israel	No reply
**Which LT4 preparations would you prescribe for**:						
Patients with variable absorption due to interfering drugs?	36 (29.3%)	4 (3.3%)	7 (5.7%)	25 (20.3%)	48 (39.0%)	3 (2.4%)
Patients with possible celiac disease, malabsorption, lactose intolerance, or intolerance to common excipients?	60 (48.8%)	1 (0.8%)	5 (4.1%)	8 (6.5%)	47 (38.2%)	2 (1.6%)
Patients with established on generic T4 who have unexplained poor biochemical control of hypothyroidism	52 (42.3%)	4 (3.3%)	6 (4.9%)	12 (9.8%)	45 (36.6%)	4 (3.3%)
Patients with poor control who is unable to separate LT4 from food / drink?	41 (33.3%)	1 (0.8%)	10 (8.1%)	17 (13.8%)	48 (39.0%)	6 (4.9%)
Patients with established generic LT4 therapy who have good biochemical control but continues to have symptoms?	43 (35.0%)	1 (0.8%)	3 (2.4%)	32 (26.0%)	42 (34.1%)	2 (1.6%)

When presented with a patient scenario of impaired absorption or inadequate control of hypothyroidism with LT4 therapy, the vast majority of respondents preferred LT4 tablets, or noted that only tablets are widely available in Israel **(**Table 2**)**. In patients treated with drugs interfering with LT4 absorption, 88.6% would use LT4 tablets (29.3% as preferred option, 39.0% because of limited availability of other formulations, 20.3% because they believed that other formulations would not make a difference). Only 9.0% stated that they would prescribe formulations other than LT4 tablets (liquid solution by 5.7% and soft gel capsules by 3.3%). For patients unable to separate LT4 from food or drink, 86.1% stated that they would continue treatment with LT4 tablets. Similarly, in patients with poor biochemical control on LT4 tablets, 88.7% stated that they would continue treatment with LT4 tablets, 3.3% would recommend soft-gel capsules, and 4.9% liquid solution.

### Patients with persistent symptoms

The prevalence of patients treated with LT4 who experience persistent symptoms despite normal serum TSH was considered to be low but not negligible by most respondents. 48% estimated that persistence of symptoms was present in less than 5% of hypothyroid patients, 30.9% reported this in 6–10% of patients, while 13% estimated that this phenomenon occurs in > 10% of patients (Table 3).

When asked if there was a trend over the past 5 years, 29.3% believed there was an increase in the amount of patients experiencing persistent symptoms despite biochemical euthyroidism, while others did not notice such a trend.

**Table 3:** Persistent hypothyroid symptoms and the role of LT3 therapy

	*n* (%)
**Some patients treated with LT4 experience hypothyroid symptoms despite normal TSH**	
**1. In your practice**,** how common is this phenomenon?**	
less than 5% of patients	59 (48.0%)
6–10%	38 (30.9%)
11–30%	15 (12.2%)
More than 30%	1 (0.8%)
Not sure	10 (8.1%)
**2. In your experience**,** what has been the trend over the past 5 years?**	
More	36 (29.3%)
Fewer	8 (6.5%)
No change	50 (40.7%)
Not sure	29 (23.6%)
**3. With regards to the etiology of this phenomenon**,** what do you think likely plays a role (one or more)**	
Inability of levothyroxine to restore normal physiology	56 (45.5%)
Psychosocial factors	82 (66.6%)
Comorbidities	32 (26%)
Chronic fatigue syndrome	28 (22.7%)
Patient unrealistic expectations	44 (35.8%)
Not sure	26 (21.1%)
**When does administration of both LT4 and LT3 may be considered?**	
For short periods, recovering from protracted hypothyroidism	11 (8.9%)
Normal TSH with symptoms suggestive of hypothyroidism	71 (57.7%)
Normal TSH with unexplained weight gain	2 (1.6%)
Should never be used due to the low quality of evidence	30 (24.4%)
No reply	9 (7.3%)

**Fig. 1:**
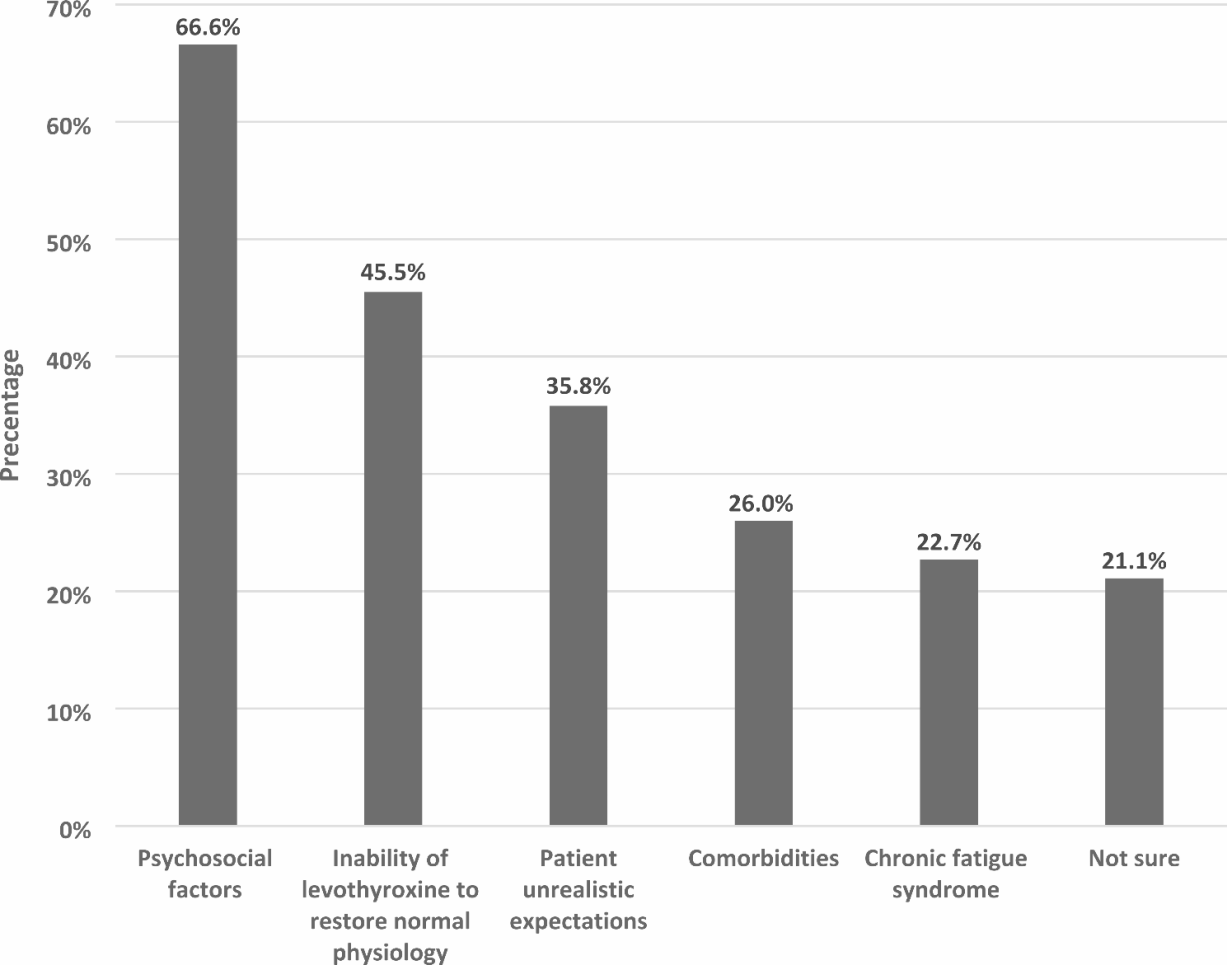
The opinion of Israeli endocrinologists on possible factors explaining persistent symptoms of hypothyroidism despite biochemical euthyroidism in patients treated with LT4

Most respondents attributed persistent symptoms to psychological factors (66.6%), together with other causes including inability of LT4 to restore normal physiology (45.5%), comorbidities (26%), chronic fatigue syndrome (22.7%), and patients’ unrealistic expectations (35.8%) (Fig. 1).

With regard to treatment of patients with persistent symptoms, 95.1% would continue LT4 tablets (34.1% noting that only tablets are available in Israel). Only 0.8% would recommend soft-gel capsules, and 2.4% would prescribe liquid solution. The treatment of choice for persistent symptoms while on LT4 tablets, was not influenced by physicians’ age, gender, or years in practice.

### Combination treatment with LT4 + LT3

Respondents were asked when the administration of both LT4 and LT3 may be considered. In patients with normal serum TSH (on LT4 treatment) with persistent symptoms, 57.7% stated that they would consider addition of LT3 to the treatment regimen, while 24.4% would never use LT4 + LT3 combination due to the low quality of evidence. Subgroup analysis demonstrated that physicians with ≤ 20 years in practice were more likely to prescribe LT3 as an addition to LT4 treatment for symptomatic patients than those with over 20 years in practice (75.8% vs. 52.4%, *p* = 0.004), whereas age and gender were not significantly associated with LT3 prescription habits. Other less common proposed indications for combination therapy included transient administration in patients recovering from protracted hypothyroidism (8.9%), or patients with unexplained weight gain (1.6%).

### Thyroid hormone use in euthyroid subjects

**Fig. 2:**
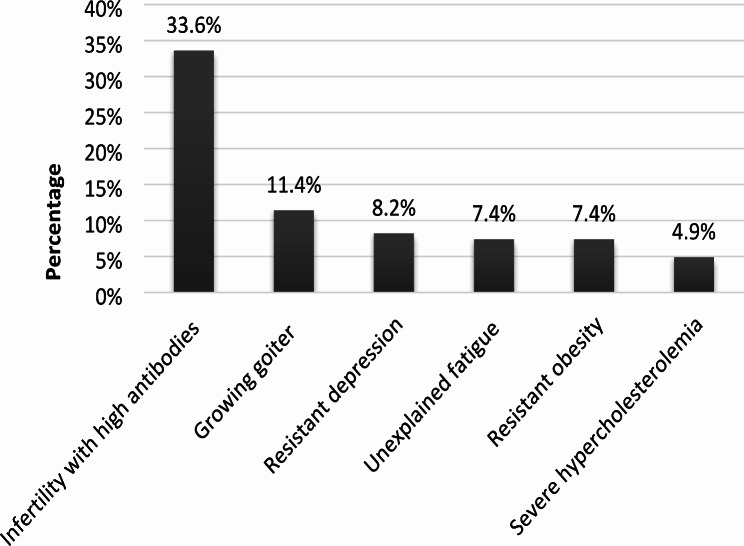
The use of thyroid hormones in euthyroid patients in Israel. Survey responders could mark none or multiple options in the questionnaire. Each bar represents the percentage of responders who marked this option

LT4 therapy in euthyroid patients was deemed as acceptable by up to 41.0% of respondents, for various possible indications, including: female infertility with high level of thyroid antibodies (33.6%), simple goiter growing over time (11.4%), depression resistant to anti-depressant medications (8.2%), unexplained fatigue (7.4%), obesity resistant to life-style interventions (7.4%), and as a complementary therapy to severe hypercholesterolemia (4.9%). Conversely, 59.0% responded that thyroid hormone treatment is never indicated for euthyroid patients (Fig. 2).

Subgroup analysis demonstrated that the decision to treat euthyroid patients was not associated with respondent demographics, including age, gender, or years in practice.

### Supplementation with selenium or iodine

Israeli endocrinologists are almost equally distributed regarding supplementations in addition to thyroid hormone replacement: 60 (48.8%) responded that supplementation with selenium or iodine could be considered, while 63 (51.2%) stated that such supplementations should not be used. Indications considered by respondents included autoimmune thyroiditis (6.5%), subclinical hypothyroidism (4.9%), or most commonly, patient’s request (37.4%). These prescription habits were not associated with respondents’ age, gender or years in practice.

## Discussion

The traditional standard of care for hypothyroid patients, namely treatment with LT4 tablets aiming for a normal serum TSH, can be challenging in daily clinical practice. Difficulties in managing hypothyroidism may be experienced in patients with non-adherence, absorption impairment, persistent symptoms despite a normal serum TSH and psychological issues. To potentially overcome some of these problems, several thyroid hormone preparations are in use in Europe, that include soft-gel capsules, liquid formulation, DTE, and LT3 formulations. While some patients benefit from changing treatment regimens, there are no official guidelines to advise how and when to use each preparation, due to missing or weak evidence, therefore prescription habits are heavily based on personal experience of clinicians and availability of formulations and treatments [3, 4].

### Thyroid hormone use and LT4 formulations

In this study, we evaluated the use of different thyroid hormone preparations by endocrinologists in Israel, where LT4 tablets are most commonly used, whereas alternative hormone preparations and LT4 formulations are not readily available. Despite the potential benefits of liquid formulations of soft-gel capsules in specific scenarios [8, 9], the clinical experience with such formulations by Israeli endocrinologists is very limited, as they are not marketed in Israel and can only be purchased via specialty pharmacies or international online pharmacies. In our study LT4 tablets were prescribed as first line treatment by 100% of respondents, which were also the most commonly prescribed preparation in scenarios where other formulations may be beneficial. This was the case in patients with impaired absorption (93.5%), patients treated with interfering drugs (88.6%), and patients with poor biochemical control on LT4 tablets (88.7%). Our experience was shared by the Danish, Belgian, Romanian and the UK THESIS surveys, where most respondents preferred tablets and did not expect a significant difference when switching to other formulations [10–13].

Our findings as for use of alternative formulations to LT4 tablets were strikingly different to those from Italy, where LT4 soft-gel capsules and LT4 liquid formulations are readily available since several years. The Italian THESIS survey [14] reported the use of liquid solution or soft-gel capsules by 81.8% of respondents when LT4 was taken together with interfering drugs, by 96.6% in patients with conditions causing impaired absorption, by 74.4% in patients with unexplained poor biochemical control and by 98.9% in patients unable to separate drug ingestion from food/drink. Also, 43.6% of Italian respondents stated that they use these formulations in hypothyroid patients with normal serum TSH and persistent symptoms [14]. The variance in use of different LT4 formulations between our and the Italian study is likely due to differences in availability of the newer formulations, marketing and different interpretations of the published evidence, which is rather weak.

### Combination therapy with LT4 + LT3 and persistent symptoms

LT4 + LT3 combination therapy remains controversial. While several randomized controlled trials and meta-analyses failed to demonstrate a benefit using this combination (10), smaller studies in patients with hypothyroid symptoms despite normal serum TSH suggested some benefit. Also, dissatisfied patients often request this therapy from treating physicians [15–19]. Israeli endocrinologists were asked in which condition LT4 + LT3 combination may be considered. Surprisingly, 57.7% stated that they would consider addition of LT3 to the treatment regimen in patients with persistent symptoms despite TSH normalization, while only 24.4% replied that LT4 + LT3 combination should not be used. This high figure may reflect increased awareness of this treatment option by respondents, as well as the availability of this combination and patient requests. However, in light of limited data regarding long term efficacy and safety, combined LT4 + LT3 treatment may be considered only in individual patients after careful consideration of risk versus benefit [6]. A recent consensus document by the American Thyroid Association (ATA), British Thyroid Association (BTA), and European Thyroid Association (ETA) formulated a guide for future clinical trials of LT4 + LT3 combination therapy, stressing the need to evaluate patients with residual symptoms, and to assess primarily patient-reported outcomes [5].

It is intriguing to acknowledge the fact that 66.6% of Israeli thyroid experts attributed persistent symptoms to psychological factors, consistents with findings observed by the UK THESIS survey [13]. In this context, it is of interest that a recent patient survey found a strong correlation between patient dissatisfaction with treatment and care and mistrust in healthcare professionals, while LT4 treatment (compared to LT3-containing regimens) was associated with a positive impact on daily living [7]. Furthermore, a high prevalence of somatization was noted among hypothyroid patients and a tendency to attribute all persistent symptoms to hypothyroidism or its treatment, suggesting that personality traits may influence the susceptibility to satisfaction with thyroid hormone treatment [20]. The dissonance between thyroid expert opinion about the etiology of persistent symptoms – considered as mostly psychological - and the frequent choice of a combination treatment, is indicative of the need for further research, not only on optimizing thyroid hormone regimens, but also seeking non-pharmacological interventions [21].

### Thyroid hormone therapy in euthyroid patients

In accordance with current guidelines, most respondents in the Israeli survey agreed that LT4 therapy is not indicated in the treatment-naïve euthyroid patients. However, 33.6% of the respondents would consider LT4 therapy in infertile women with high level of thyroid antibodies. This is in agreement with reports from the French, the Romanian and the Finnish surveys (31.7%, 36.4%, and 30%, respectively), but is slightly lower than in other countries in the THESIS survey, namely 48.5% of the Spanish, 42.1% of the Danish, and considerably lower compared to the German (63%) and the Polish (63.4%) surveys [10, 12, 22–26].

The above practices are consistent with the 2012 ETA guidelines [27], suggesting that treatment with LT4 might be considered in some euthyroid TPOAb-positive women undergoing fertility treatment, in whom serum TSH is within the upper normal reference range (i.e., 2.5–4.0 mIU/L) in order to ensure euthyroidism in case of pregnancy. This approach is at variance with recent data demonstrating that LT4 treatment neither promotes conception nor reduces the risk of pregnancy complications in such individuals [28].

With respect to thyroid hormone treatment for euthyroid goitre, in view of the absence of long-term efficacy after treatment withdrawal, and evidence that prolonged TSH suppression is associated with increased somatic and psychiatric morbidity as well as increased mortality, such treatment is discouraged by recent guidelines [29–34]. The THESIS studies have revealed substantial variation between countries, with a relatively low proportion (11.4%) of Israeli and UK (9.3%) [13], respondents advocating thyroid hormone therapy for simple growing goiter, in contrast to other European countries (Germany 57.1% [25], Romania 39.4% [12], France 40.2% [22], Poland 40.3% [24]). These differences in responses may relate to clinician practices being influenced by the prevalence of iodine deficiency, long established habits, presence or absence of national guidelines and clinician access to educational material.

### Strengths and limitations

Strengths of our study include the standardized questionnaire across European countries, and the high response rate to the survey. On the other hand, our study is limited by its design of an online survey, where specific questions are formulated and respondents participate by clicking on offered options, and performed during the COVID-19 pandemic restrictions [13]. This design may not fully dissect the complexity of the topic, and participants may not be able to explain the reasons for their choices. Second, the survey was conducted among Israeli endocrinologists, and did not include primary care physicians. While in our system many patients with hypothyroidism are treated by primary care physicians, a large proportion of patients have an endocrinologist consultation at least once during their follow-up, and formulations other than LT4 are prescribed exclusively by endocrinologists. Third, the answers in the survey were presented as binary yes or no answers, and therefore may have may have missed some scaled responses, as this was an opinion survey. Last, some relevant aspects of thyroid hormone use were not addressed in this survey, including hypothyroid emergencies, treatment in pregnancy, or treatment in elderly patients.

## Conclusions

In conclusion, LT4 tablets are the treatment of choice by Israeli endocrinologists for most clinical scenarios, including patients with impaired absorption or persistent symptoms of hypothyroidism despite biochemical euthyroidism. Soft-gel capsules and liquid solutions, which are not widely available in Israel, are infrequently recommended as compared with other European countries.

Combination therapy with LT4 + LT3 is widely considered in Israel for patients treated with LT4 with persistent symptoms despite biochemical euthyroidism, although this therapeutic strategy contrasts with published evidence showing lack of superiority of combination treatment compared to LT4 alone. Moreover, a significant minority of Israeli thyroid experts would consider LT4 treatment in specific euthyroid patient groups, a practice which is largely not based on evidence, but also noted by other national THESIS surveys.

The wide variability on the use of thyroid hormones across countries is concerning and indicative of the need for more data where evidence is lacking and better implementation of guidelines where the evidence is adequate.

## Data Availability

No datasets were generated or analysed during the current study.

## Abbreviations

LT4: Levothyroxine
DTE: Desiccated thyroid extract
ETA: European Thyroid Association
THESIS: Treatment of Hypothyroidism in Europe by Specialists: An International Survey
IES: Israeli Endocrine Society
HMOs: Israeli health maintenance organizations
